# Sarcoid-like reaction in patients with malignant tumors: Long-term clinical course and outcomes

**DOI:** 10.3389/fmed.2022.884386

**Published:** 2022-08-17

**Authors:** Jin-Young Huh, Do Sik Moon, Jin Woo Song

**Affiliations:** Department of Pulmonary and Critical Care Medicine, Asan Medical Center, University of Ulsan College of Medicine, Seoul, South Korea

**Keywords:** sarcoidosis, neoplasms, granuloma, mortality, progression-free survival

## Abstract

**Background:**

The development of non-caseating epithelioid cell granulomas in cancer patients who do not fulfill the systemic sarcoidosis criteria is termed sarcoid-like reaction (SLR). Little is known about this condition's natural course and impact on the prognosis of malignancy. We aimed to investigate the natural course and prognostic value of cancer-associated SLR.

**Methods:**

Clinical data were retrospectively analyzed in 32 patients with biopsy-proven cancer-associated SLR. Among patients with non-small cell lung cancer (NSCLC), SLR cases (*n* = 8) were matched with non-SLR cases (*n* = 78) for survival analysis.

**Results:**

Among the included patients, the mean age was 59.7 years, and 68.8% were female. The median follow-up period was 35.6 months [interquartile range (IQR): 14.0–61.4 months]. Of all the included malignancies (*n* = 32), breast cancer (25.0%) and NSCLC (25.0%) were the most common, with stage I being the most frequent tumor stage (59.4%). During follow-up, SLR progression to overt sarcoidosis was not observed. In the 28 patients with available follow-up computed tomography images (median interval: 24.9 months; IQR: 14.4–41.7), 4 patients received corticosteroids (*n* = 4), resulting to a decrease of SLR lesions. Meanwhile, among those who did not receive treatment (*n* = 24), the extent of SLR decreased or did not change in 85.7% of them, whereas 3.6% had increased SLR extent. Furthermore, among patients with NSCLC, SLR was not associated with overall survival [hazard ratio (HR) = 1.28, 95% confidence interval (CI): 0.02–67.71, *P* = 0.882] and recurrence of malignancy (HR = 1.27, 95% CI 0.21–7.51, *P* = 0.793) in the Cox proportional hazard regression model.

**Conclusions:**

During the follow-up of cancer-related SLR, we found no further evidence for systemic sarcoidosis, and most of the lesions decreased or did not change. Development of SLR was also not associated with overall survival or disease-free survival in patients with NSCLC.

## Introduction

Sarcoid-like reaction (SLR) is defined as the development of non-caseating epithelioid cell granulomas in patients who do not meet the criteria for systemic sarcoidosis (1). Its occurrence is known to be linked with diverse conditions, including malignancies (2–4), infections (5, 6), and exposures to inorganic substances or certain drugs (7–9). Studies have suggested that the pathogenesis of SLR is attributed to diverse antigens that are coupled with genetic susceptibility, consequently triggering T cell mediated immune responses, which then leads to the formation of non-necrotizing epithelioid cell granulomas (10).

Although various types of malignancies, including lymphoma (11, 12), breast cancer (13, 14), stomach cancer (15, 16), colon cancer (17, 18), and lung cancer (19, 20), have been reported to be associated with SLR development, its clinical significance in cancer patients remains unclear. In previous studies (14, 21), SLR has been associated with better cancer outcomes. In particular, Chen et al.'s study in five breast cancer patients with SLR showed that all patients were disease-free during the median follow-up of 6 years following curative resection (14). Similarly, Steinfort et al. reported that among 24 patients with early-stage (stage I) non-small cell lung cancer (NSCLC), no recurrence after lobectomy was observed in those with SLR (*n* = 8), whereas a recurrence rate of 44% was found in those without SLR (*n* = 16) (21). However, contradicting findings have also been reported; Tomimaru et al., for one, reported that among 1,733 lung cancer patients who underwent surgical resection, no significant difference in the overall survival (77.7 vs. 75.2%, *P* = 0.8227) was found between patients with (*n* = 22) and without SLR (*n* = 1,711) (19). Given all these findings, there are still uncertainties regarding impact of SLR on the prognosis of malignancy, and notably, the clinical course of SLR has not been addressed in previous studies. Therefore, we aimed to investigate the clinical course and prognostic value of SLR in patients with malignancies.

## Materials and methods

### Study population

**Figure 1:**
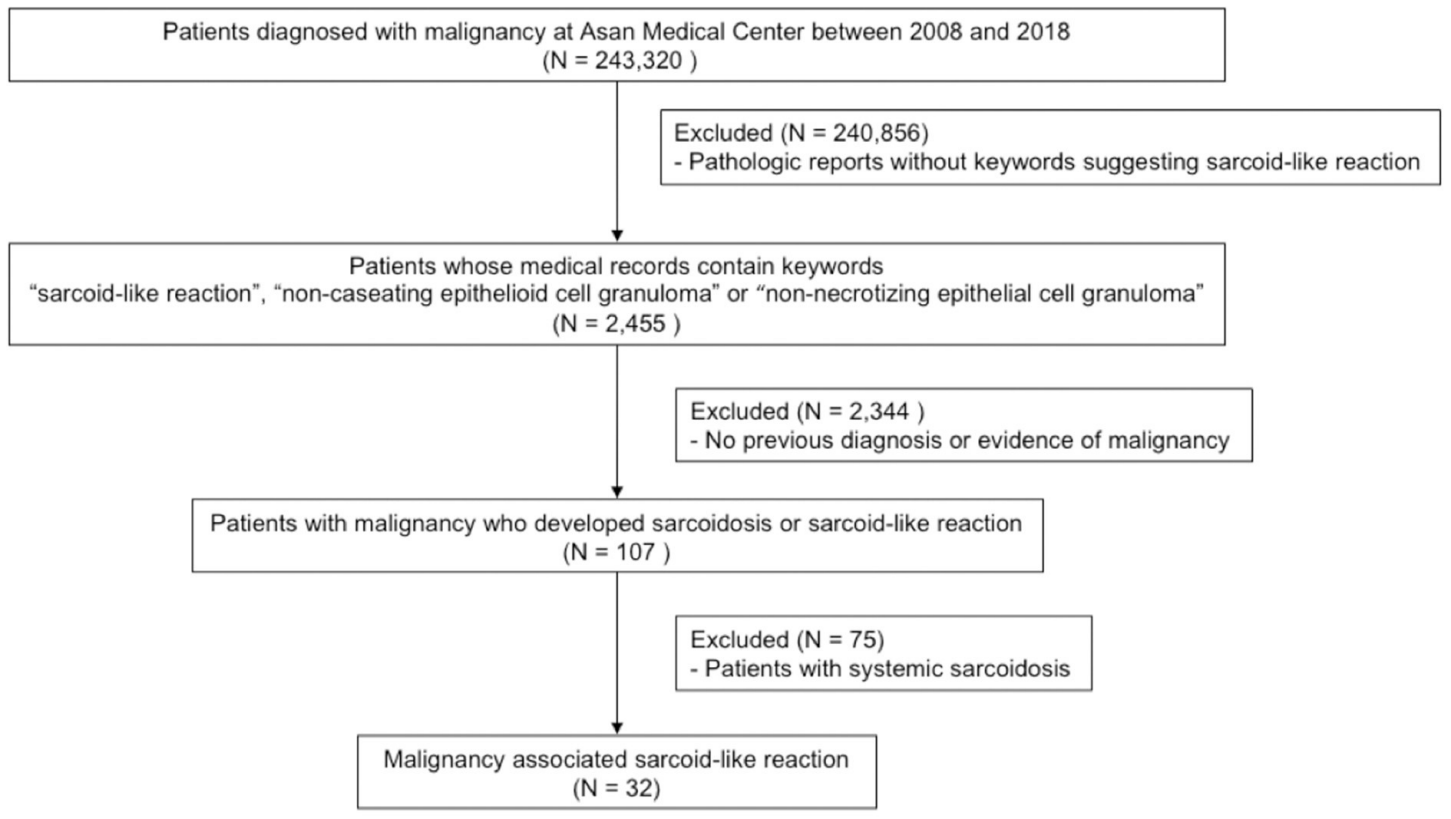
Flowchart of the study.

Adult patients with ICD-10 codes for malignant neoplasm (C00-C97) identified between 2008 and 2018 at the Asan Medical Center, Seoul, Republic of Korea (*n* = 243,320) were screened for this study. Among them, 2,455 cases with medical records containing the following keywords were selected: “sarcoid-like reaction,” “non-caseating epithelial cell granuloma,” or “non-necrotizing epithelial cell granuloma.” After reviewing the pathologic reports of these cases, 2,344 patients were excluded since no previous diagnoses or evidence of malignancy were provided at the time of biopsy. From the remaining 107 patients, those diagnosed with overt sarcoidosis (*n* = 75) were excluded. Overt sarcoidosis was defined by evidence of two or more organ involvements at the time of biopsy (22). Patients' past medical history, ophthalmologic tests, electrocardiograms, echocardiography, Holter monitoring, laboratory examinations, including serum creatinine, serum alkaline phosphatase and complete blood cell count, and urine calcium levels were reviewed (Supplementary Table 1). Finally, a total of 32 patients with malignancy associated SLR were included in this study (Figure 1).

The study protocol was approved by the Institutional Review Board of the Asan Medical Center (IRB No. 2019-1015), and the requirement for informed consent was waived due to the retrospective nature of the analysis.

### Data collection

The clinical and survival data of all the included patients were retrospectively collected from their medical records and/or the National Health Insurance records of Korea. Spirometry, diffusing capacity of the lung for carbon monoxide, and total lung capacity were measured according to the ERS/ATS recommendations (16, 17), and the results were expressed as percentages of the normal predicted values.

Data from follow-up visits or hospitalizations were reviewed to determine malignancy recurrence. To evaluate the clinical course of SLR, baseline and the follow-up computed tomography (CT) images [median interval: 24.9 months, interquartile range (IQR): 14.4–41.7 months] were compared side by side by two readers (JYH and DSM), wherein disagreements were resolved *via* consensus. For each subsequent CT scan reading, findings were categorized into “improved,” “unchanged,” or “aggravated.” Specifically, an SLR lesion was categorized as “improved” if the sum of lengths of the lesions decreased by 30% or more, whereas an “aggravated” SLR lesion was defined if the sum of lengths of the lesions increased by 20%, which is based on the modified RECIST criteria (23). Lesions without changes or with changes that did not belong to either the “improved” or “aggravated” categories were defined as “unchanged” SLR lesions.

### Case-control matching in NSCLC

For the evaluation of the impact of SLR on the prognosis of patients with NSCLC, overall survival and disease-free survival were compared between patients with and without SLR. NSCLC patients without SLR were selected from the Center for Cancer Data Management at Asan Medical Center. Following selection, case-control matching (1:10) was performed to adjust for differences in baseline characteristics, including age, sex, T stage, N stage, the time of cancer diagnosis, and treatment modality.

### Statistical analysis

All values are presented as means ± standard deviations for continuous variables or as percentages for categorical variables. Survival was assessed using the Kaplan–Meier survival analysis, and differences between groups were evaluated using the log-rank test. Furthermore, the Cox proportional-hazards model was applied to evaluate impact of SLR on overall survival and disease-free survival. All statistical analyses were performed using the R Statistical Software (version 4.0.3; R Foundation for Statistical Computing, Vienna Austria), and a *P*-value <0.05 was considered to be statistically significant (two-tailed).

## Results

### Baseline characteristics

Among the included patients in this study, the mean age was 59.7 years, and 68.8% were female (Table 1). The median follow-up period from the date of SLR diagnosis was 35.6 months (IQR: 14.0–61.4 months). Notably, the mean angiotensin-converting enzyme (ACE) level was 38.4 U/L (*n* = 14), which was elevated (normal range: 7.5–53.0 U/L) in 28.6% (4/14) of them.

**Table 1:** Baseline characteristics of the study population.

**Characteristics**	
Patient number	32
Age, years	59.7 ± 11.7
Female sex	22 (68.8)
Smoking status
Current smoker	2 (6.2)
Ever-smoker	8 (25.0)
Never-smoker	22 (68.8)
Angiotensin-converting enzyme, U/L	38.4 ± 21.8
Pulmonary function test
FVC, % predicted	84.7 ± 17.8
DLCO, % predicted	77.4 ± 16.7
TLC, % predicted	93.6 ± 20.8
6-minute walk test
Distance, meter	490.1 ± 49.8
Lowest SpO_2,%_	95.6 ± 2.4
BAL fluid analysis
WBC, /mm^3^	120.8 ± 112.3
Neutrophil, %	1.8 ± 1.6
Lymphocyte, %	21.6 ± 18.9
Site of sarcoid-like reaction
Mediastinal lymph node	21 (65.6)
Neck lymph node	7 (21.9)
Lung parenchyma	2 (6.3)
Axillary lymph node	1 (3.1)
Intraabdominal lymph node	1 (3.1)

Data are presented as means ± standard deviations, or as numbers (%), unless otherwise indicated. FVC, forced vital capacity; DLCO, diffusing capacity of the lung for carbon monoxide; TLC, total lung capacity; SpO_2_, saturation of oxygen; BAL, bronchoalveolar lavage; WBC, white blood cell.

For all patients, SLR developed in various types of cancer. Among them, breast cancer (25.0%) and NSCLC (25.0%) were the most common, which was followed by colon cancer (18.8%) (Table 2). The median interval between cancer and SLR diagnoses was 3.8 months (IQR: 0.7–30.5 months), with most patients (81.3%) reporting SLR occurrence within 3 years after cancer diagnosis (Supplementary Figure 1). Regarding tumor staging at the time of SLR diagnosis, stage I was the most frequently diagnosed stage (59.4%, 19/32), while only one patient had stage IV disease (Table 2). Also, in terms of the treatment of malignancy, 26 (81.3%) underwent surgical resection, 4 (12.5%) received chemotherapy, 1 (3.1%) had concurrent chemoradiotherapy, and 1 (3.1%) was treated with radiotherapy. No patients were treated with immune check point inhibitors. Furthermore, 93.8% of the patients presented with lymphadenopathy (mediastinum: 65.6%, neck: 21.9%, axilla: 3.1%, intrabdomen: 3.1%), and 6.3% had lung lesions (Table 1). For mediastinal lymph nodes, endobronchial ultrasound-guided transbronchial needle aspiration was performed and, for lymph nodes at other sites, ultrasound-guided needle biopsy was done. The samples of the lung parenchymal lesions were acquired with percutaneous lung biopsy.

**Table 2:** Types and stage of the malignancy in patients with sarcoid-like reaction.

**Characteristics**	
Patient number	32
Type of cancer
Breast cancer	8 (25.0)
Non-small cell lung cancer	8 (25.0)
Colon cancer	6 (18.8)
Non-Hodgkin's lymphoma	3 (9.4)
Gastric cancer	2 (6.3)
Thyroid cancer	2 (6.2)
Hodgkin's lymphoma	1 (3.1)
Prostate cancer	1 (3.1)
Cervix cancer	1 (3.1)
Stage of cancer
I	19 (59.4)
II	6 (18.8)
III	6 (18.8)
IV	1 (3.1)

Data are presented as numbers (%), unless otherwise indicated.

### Clinical outcomes of malignancy

During follow-up, two patients with SLR (6.3% of total patients) died. Specifically, one patient with Hodgkin lymphoma died due to an acute exacerbation of combined idiopathic pulmonary fibrosis, whereas the other patient with NK/T cell lymphoma died from hemophagocytic lymphohistiocytosis progression. Moreover, malignancy recurrence following curative treatment was observed in one patient with SLR (3.1%). In this case, the patient with NSCLC (adenocarcinoma, stage IB) underwent surgical resection for curative treatment; however, brain metastasis occurred 35 months after SLR diagnosis. On the other hand, no evidence of disease progression or recurrence was observed in the remaining 29 patients with SLR.

**Figure 2:**
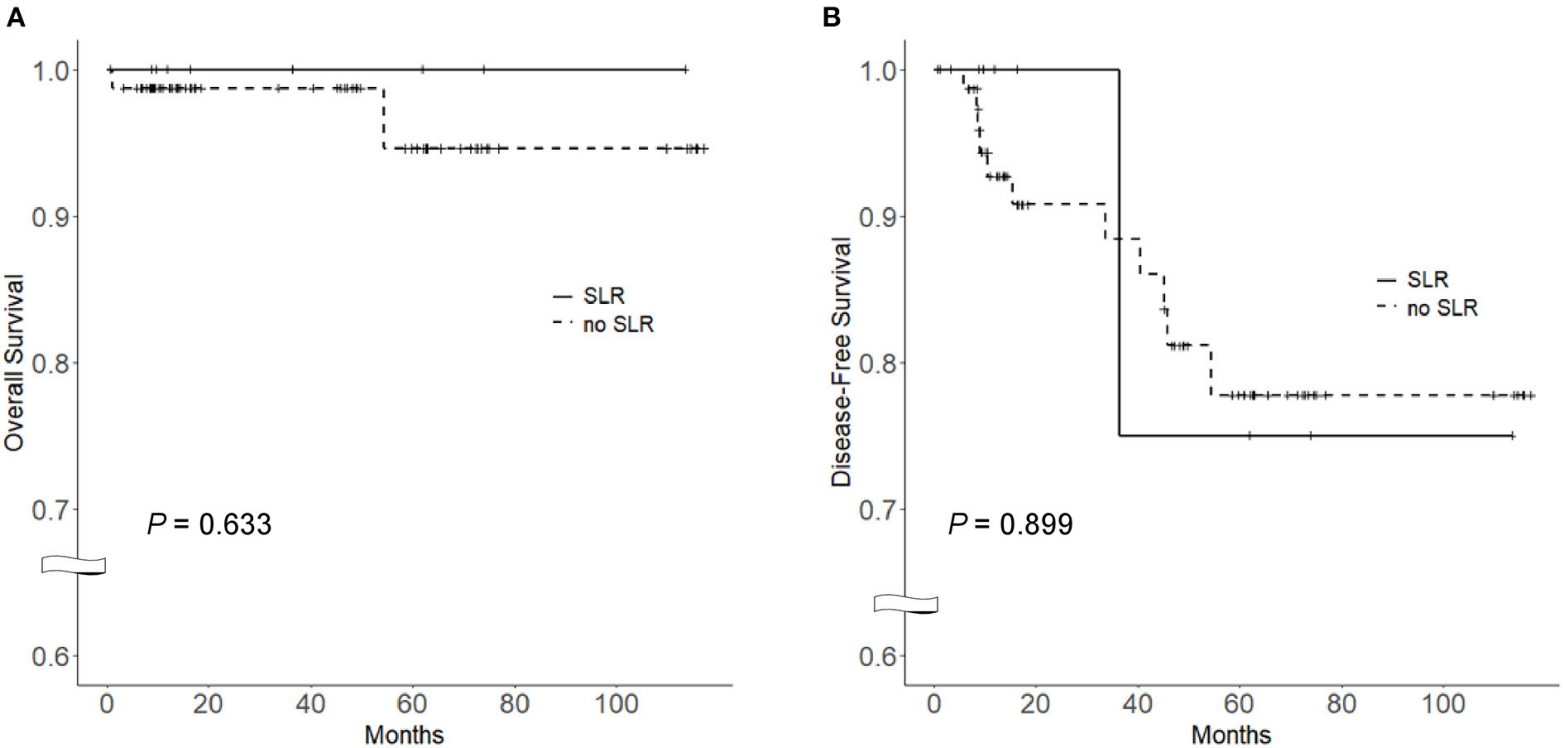
Comparison of the Kaplan–Meier survival curves between SLR and non-SLR groups among NSCLC patients. **(A)** Overall survival curve, **(B)** Disease-free survival curve. Log-rank test was used for the analysis. SLR, sarcoid-like reaction; NSCLC, non-small cell lung cancer.

To compare survival between NSCLC patients with (*n* = 8) and without SLR, a control cohort was selected from the Asan Medical Center database (*n* = 79). Baseline clinical characteristics and outcomes between the two groups are shown in Table 3. No deaths were reported in the SLR group, while two patients (2.5%) died in the control group (*P* > 0.99). One patient (12.5%) in the SLR group had recurrence of NSCLC, whereas 11 patients (13.9%) in the control group reported disease recurrence *P* > 0.99 as presented in Table 2. No significant differences were observed in the overall survival [100.0% (SLR group) vs. 94.6 (control group), *P* = 0.633] (Figure 2A) and disease-free survival (75.0 vs. 77.8%, *P* = 0.899) between both groups (Figure 2B). In a multivariate Cox proportional-hazards model adjusted for age, sex, cell type and treatment modality, SLR was not associated with overall survival (hazard ratio = 1.28; 95% confidence interval, 0.02–67.71; *P* = 0.882) and disease-free survival (hazard ratio = 1.27; 95% confidence interval, 0.21–7.51; *P* = 0.793) (Supplementary Table 2).

**Table 3:** Comparison of baseline characteristics and clinical courses between SLR and non-SLR groups among NSCLC patients.

**Characteristics**	**SLR**	**No-SLR**	***P*-value**
Patient number	8	79	
Age, years	68.6 ± 6.0	69.0 ± 6.1	0.877
Male sex	4 (50.0)	39 (49.4)	>0.99
Cell type			>0.99
Adenocarcinoma	7 (87.5)	69 (87.3)	
Squamous cell carcinoma	1 (12.5)	10 (12.7)	
Treatment			>0.99
Surgical resection	7 (87.5)	69 (87.3)	
Radiotherapy	1 (12.5)	10 (12.7)	
Chemotherapy	0 (0.0)	0 (0.0)	
Median follow-up period	33.2 (11.6–64.8)	18.5 (11.6–62.7)	0.831
Recurrence	1 (12.5)	11 (13.9)	>0.99
Death	0 (0.0)	2 (2.5)	>0.99
Disease-free survival, months	26.3 (11.2–41.5)	17.4 (10.3–38.0)	0.814

Data are presented as means ± standard deviations, numbers (%), or as medians (interquartile range), unless otherwise indicated.

SLR, sarcoid-like reaction; NSCLC, non-small cell lung cancer.

### Clinical outcomes of SLR

**Figure 3:**
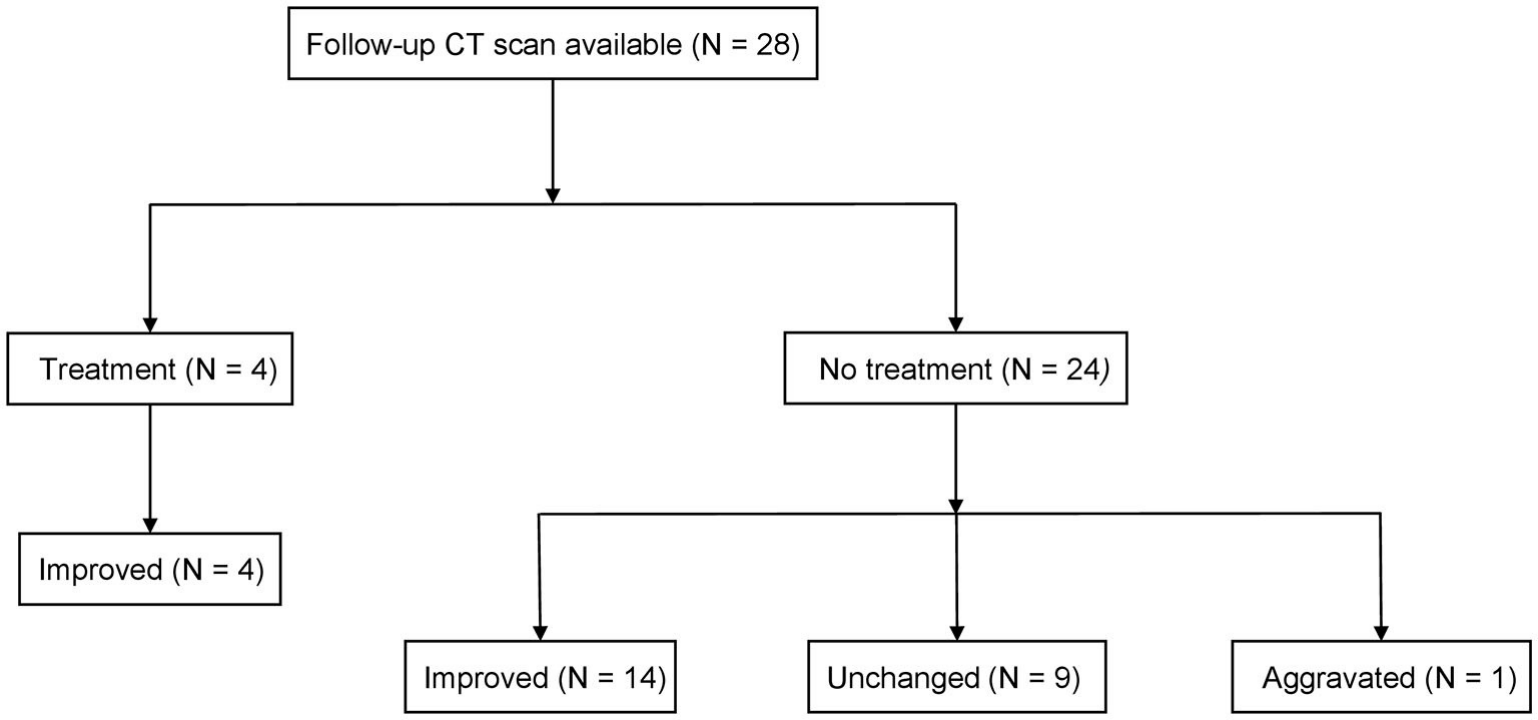
Clinical course of the patients with SLR. Patients who received treatment were given prednisolone with a mean dose of 28.8 mg. Improvements were defined as a decrease in the sum of the lengths of SLR lesions by 30% or more. Aggravation was defined as an increase in the sum of the lengths of SLR lesions by 20% or more. SLR, sarcoid-like reaction.

Among the 32 included patients, follow-up CT images were available in 28 patients, which were analyzed to evaluate the clinical course of SLR. Notably, four patients 14.3% were treated with corticosteroids [initial mean dose: 28.8 ± 2.5 mg of prednisolone, the median treatment period: 20.1 months (IQR: 15.9–23.7 months)] at the attending physician's discretion; one patient with co-existing chronic obstructive pulmonary disease received corticosteroid due to progressive dyspnea, and the other three patients were treated with corticosteroid to further confirm that the lesions were due to SLR and not metastasis. All four patients had an improvement in all SLR lesions (100%). Meanwhile, in the untreated group (*n* = 24), the outcomes were categorized as “improved” in 58.3%, “unchanged” in 37.5%, and “aggravated” in 4.2% (Figure 3). There was no further evidence of systemic sarcoidosis during the follow-up.

## Discussion

In this study, we identified 32 biopsy-confirmed SLR patients with various types of malignancies. At the time of SLR diagnosis, the most common underlying cancers were breast cancer and NSCLC, with stage I as the most common tumor staging. During follow-up, most of SLR lesions improved or did not progress in treatment-naïve cases, whereas SLR lesions improved in all treatment cases. We found no further evidence of systemic sarcoidosis during the follow-up. Furthermore, overall survival and disease-free survival were similar between the SLR and non-SLR groups of NSCLC patients.

The patients included in this study were mostly in the early-stage of their malignancy (stage I in 59.4%). This was similar to the observations in Steinfort et al.'s study of 187 NSCLC patients, wherein they reported that all eight cases (4.3%) with SLR were stage I (20). Murthi et al.'s multicenter study including 133 cancer-associated SLR patients also reported that stage I was the most common tumor staging (38.3% of 131 patients with available data for cancer stages) (2). The pathogenesis of SLR in malignancy has been postulated by previous studies to be caused by immune responses to malignancy-related antigens involving dendritic cells and T cells (2, 10). Vimentin, a type III intermediate filament that forms the cytoskeletal microtubules and microfilaments (24), has been a suspected culprit antigen for SLR in cancer patients (2). While this is plausible, vimentin is not expressed universally and its expression has been associated with worse prognosis or advanced disease (25, 26). Diversity of cancer associated with SLR, and findings of more frequent early-stage cancers suggest further investigation for the causative antigen is required and we may even find that not a single antigen is responsible for all cases of cancer-related SLR. In fact, the T cell response to the antigen may be the key to impact of SLR on the clinical course. The presence of tumor-infiltrating T cells has been known to be associated with better prognosis (27), and it has been suggested that SLR might be associated with potential antitumor response, leading to cancer progression inhibition (21).

In the comparison between NSCLC patients with and without SLR, no association was found between SLR and the prognosis of NSCLC patients. Our results showed that no deaths occurred in the SLR group, whereas two deaths were found in the non-SLR group (mortality rate: 0.0 vs. 2.5%, *P* > 0.99). Tomimaru et al. showed similar results in their study of lung cancer patients who underwent surgical resection, reporting that the 3-year (85.2 vs. 82.1%) and 5-year survival rates (77.7 vs. 75.2%) were similar between the SLR (*n* = 22) and non-SLR (*n* = 1,711) groups (19). However, in other previous studies, SLR was associated with better outcomes in NSCLC patients (2, 19, 28). Dagaonkor et al.'s study, among 127 lung cancer patients who underwent surgery, reported that the survival rate was higher (3-year survival rate: 21.1 vs. 6.5%, *P* = 0.06) in those with SLR (*n* = 19) than those without SLR (*n* = 108) (28). Murthi et al.'s study of 133 patients with various cancer-related SLRs (lung cancer, *n* = 30) also showed that survival was significantly better (odds ratio, 0.223; 95% confidence interval, 0.079–0.632; *P* = 0.005) in the SLR group (*n* = 46) than in the non-SLR group (*n* = 134) (2). Furthermore, Steinfort et al. showed that among NSCLC patients, disease-free survival was better in the SLR group (*n* = 8), as compared to the non-SLR group (*n* = 16) (100 vs. 56%, *P* = 0.044) (21). This discrepancy between our study and previous investigations may be due to small number of patients in our study's SLR group. Additionally, our controls were matched based on age, sex, T stage, N stage, and the time of NSCLC diagnosis, whereas in other studies, the controls were either unmatched (2, 28) or matched regardless of the time of diagnosis (21).

Regarding the clinical course, most of the SLR lesions either improved or did not progress during follow-up; this was consistent with the results of previous studies (29, 30). Lau et al.'s study of 11 patients with SLR (associated with malignancy, hepatitis C, or medication) showed that the extent of SLR lesions decreased in 81.8%, whereas 18.2% reported no changes during the median follow-up of 10.6 months (IQR: 6.5–32.6 months) (29). Kaneko et al. also reported that among 14 patients who developed SLR following malignant tumor treatment, SLR regressed in 78.6%, remained unchanged in 7.1%, and worsened in 14.3% of the patients (30). In our study, the only patient whose CT findings were “aggravated” presented with mediastinal lymph node enlargements as a manifestation of SLR. In addition, at the time of the patient's last chest CT scan, pneumonia was also reported, possibly contributing to the enlargement of the lymph nodes. Given all these findings, our study and previous studies suggest a favorable clinical course in patients with SLR. Furthermore, all patients treated with prednisolone in our study showed significant improvements, suggesting the effectiveness of corticosteroid therapy for SLR.

Interestingly, the level of ACE was not elevated in most of our patients, with only 28.6% of patients reporting an elevated ACE level. The ACE is secreted by epithelioid and giant cells, which becomes elevated among patients with epithelioid and giant cell-containing granulomas (31). In particular, a non-caseating granuloma with epithelioid and giant cells is a pathologic hallmark of sarcoidosis (32), and SLR is also characterized by epithelioid granulomas (21). Despite this pathologic linkage, previous studies have shown that the mean level of ACE was not elevated in most patients with SLR (2, 3). Murthi et al., for one, reported on the ACE levels of 54 patients from 133 cancer-related SLR patients, showing elevated ACE levels in 15.7% of them (2). Another case-control study by Pastre et al. regarding cancer patients with SLR reported ACE level elevation in 50.0% (16/32) of them (3). Given all these findings, the studies suggest that ACE levels can be variable in SLR.

Several limitations of this study should be noted. First, as we restricted patients to those who underwent biopsy at a single center, selection bias might have affected the results. However, the clinical features of our patients were comparable to those in previous studies (2, 28). Second, the number of patients included in our study was small, consequently confounding statistical analysis and data interpretation. However, our results suggested a favorable clinical course of SLR in cancer patients despite the small sample size. Third, prognosis was only evaluated in NSCLC, and the included number of patients was very small. Nevertheless, this is one of the only few reports in the literature of the prognostic value of SLR among NSCLC patients and the relatively low incidence can make it difficult to conduct large-scale prospective studies of this subject. Lastly, chemotherapy for cancer has been associated with granulomas in previous reports (8, 9), and some of the patients in our study were exposed. Nonetheless, only about 15% were treated with chemotherapy before the diagnosis of SLR and the medications did not include immune check point inhibitors, which are commonly related. Despite these limitations, investigations on the long-term clinical course and prognostic impact of SLR were strengths of the study.

In conclusion, among cancer-associated SLR patients, early-stage breast cancer and NSCLC were the most common underlying malignancies. Our findings showed no further evidence of systemic sarcoidosis after initial evaluation, wherein most of the SLR lesions decreased or did not change during follow-up. Furthermore, SLR development may not be associated with the prognosis of NSCLC patients.

## Data availability statement

The original contributions presented in the study are included in the article/Supplementary material, further inquiries can be directed to the corresponding author/s.

## Ethics statement

The studies involving human participants were reviewed and approved by Institutional Review Board of Asan Medical Center. Written informed consent for participation was not required for this study in accordance with the national legislation and the institutional requirements.

## Author contributions

JWS, DM, and J-YH contributed to conception and design of the study. DM organized the database. DM and J-YH performed the statistical analysis. J-YH wrote the first draft of the manuscript. All authors contributed to manuscript revision, read, and approved the submitted version.

## Funding

This study was supported by a grant from the Basic Science Research Program (NRF-2022R1A2B5B02001602) and the Bio & Medical Technology Development Program (NRF-2022M3A9E4082647) of the National Research Foundation of Korea (NRF) funded by the Ministry of Science & ICT, Republic of Korea.

## Conflict of interest

The authors declare that the research was conducted in the absence of any commercial or financial relationships that could be construed as a potential conflict of interest.

## Publisher's note

All claims expressed in this article are solely those of the authors and do not necessarily represent those of their affiliated organizations, or those of the publisher, the editors and the reviewers. Any product that may be evaluated in this article, or claim that may be made by its manufacturer, is not guaranteed or endorsed by the publisher.
