# Supplementary material for: Sarcoid-like reaction in patients with malignant tumors: Long-term clinical course and outcomes
Source: Front Med (Lausanne). 2022 Aug 17;9:884386. doi: 10.3389/fmed.2022.884386 (PMC9433121; doi:10.3389/fmed.2022.884386)
Supplement: Supplementary file 1 [file Data_Sheet_1.docx]

**Supplementary Material**

**Sarcoid-like reaction in patients with malignant tumors: long-term clinical course and outcomes**

Jin-Young Huh, MD^1†^, Do Sik Moon, MD^1†^, Jin Woo Song, MD, PhD^1^

^1^Department of Pulmonary and Critical Care Medicine, Asan Medical Center, University of Ulsan College of Medicine, Seoul, Republic of Korea

^†^These authors have contributed equally to this work

**FIGURE LEGENDS**

Supplementary Figure 1. Number of patients according to the interval from cancer diagnosis to sarcoid-like reaction diagnosis.

The X-axis represents the interval from cancer diagnosis to sarcoid-like reaction diagnosis. The Y-axis represents the number of patients.


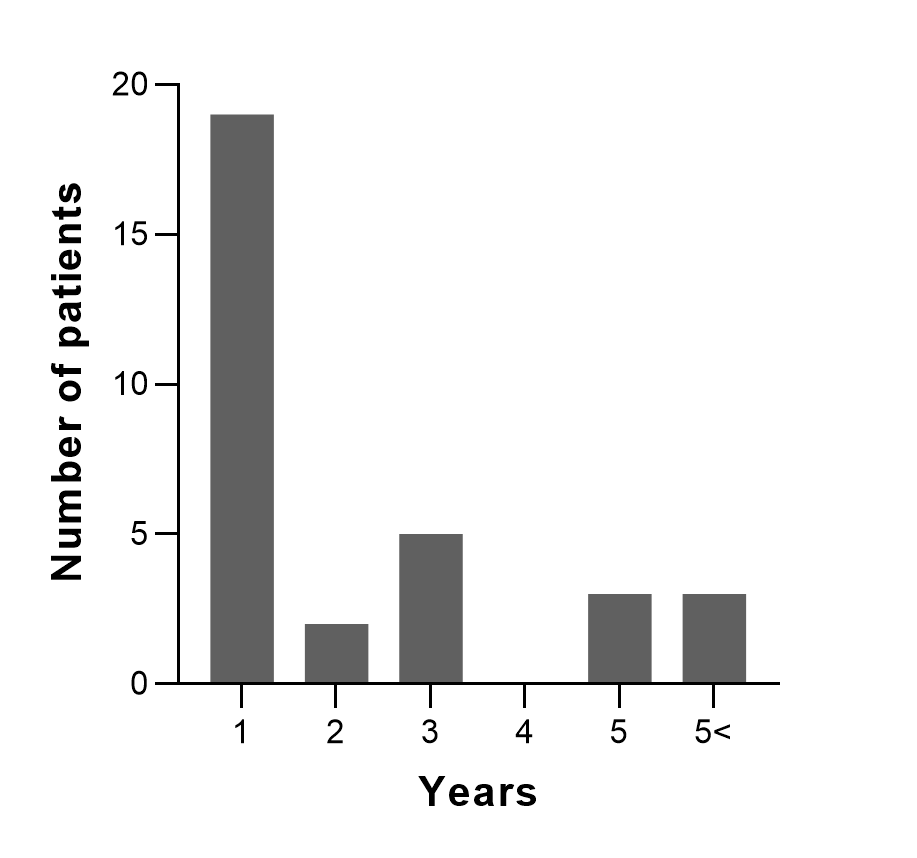


Supplementary Table 1. Examinations to exclude presence of overt sarcoidosis in 32 patients included in this study.

| Examinations |  |
| --- | --- |
| Patient number | 32 |
| Laboratory examinations^*^ | 32 (100) |
| Electrocardiogram | 32 (100) |
| Echocardiography | 21 (65.6) |
| Ophthalmologic evaluation | 19 (59.4) |
| Urine calcium | 6 (18.8) |
| Holter monitoring | 4 (20.0) |

Data are presented as numbers (%).

^*^Laboratory examinations include serum creatinine, serum alkaline phosphatase and complete blood cell count.

Supplementary Table 2. Multivariate cox regression analysis of mortality and recurrence in patients with non-small cell lung cancer.

|  | Mortality | |  | Recurrence | |
| --- | --- | --- | --- | --- | --- |
|  | Hazard ratio | *P* value |  | Hazard ratio | *P* value |
| Age | 0.790 | 0.205 |  | 1.056 | 0.402 |
| Female | 2.451 | 0.553 |  | 1.874 | 0.427 |
| Operation | 0.087 | 0.381 |  | 0.117 | 0.053 |
| SqCC | 1.653 | 0.826 |  | 0.529 | 0.529 |
| SLR | 1.284 | 0.882 |  | 1.265 | 0.793 |

SLR, sarcoid-like reaction; SqCC, squamous cell carcinoma
